# Facilitating Factors and Barriers to the Use of Emerging Technologies for Suicide Prevention in Europe: Multicountry Exploratory Study

**DOI:** 10.2196/mental.7784

**Published:** 2018-01-24

**Authors:** Juan-Luis Muñoz-Sánchez, Carmen Delgado, Andrés Sánchez-Prada, Esther Parra-Vidales, Diego de Leo, Manuel Franco-Martín

**Affiliations:** ^1^ Department of Psychiatry Zamora Hospital Zamora Spain; ^2^ Faculty of Psychology Universidad Pontificia de Salamanca Salamanca Spain; ^3^ Intras Foundation Zamora Spain; ^4^ Australian Institute for Suicide Research and Prevention Griffith University Queensland Australia

**Keywords:** suicide, prevention, technology

## Abstract

**Background:**

This study provides an analysis on the use of emerging technologies for the prevention of suicide in 8 different European countries.

**Objective:**

The objective of this study was to analyze the potentiality of using emerging technologies in the area of suicide prevention based on the opinion of different professionals involved in suicide prevention.

**Methods:**

Opinions of 3 groups of stakeholders (ie, relevant professionals in suicide field) were gathered using a specifically designed questionnaire to explore dimensions underlying perceptions of facilitating factors and barriers in relation to the use of emerging technologies for suicide prevention.

**Results:**

Goal 1 involved facilitating factors for the use of emerging technologies in suicide prevention. Northern European countries, except for Belgium, attach greater relevance to those that optimize implementation and benefits. On the other hand, Southern European countries attach greater importance to professionally oriented and user-centered facilitating factors. According to different stakeholders, the analysis of these facilitating factors suggest that professionals in the field of social work attach greater relevance to those that optimize implementation and benefits. However, professionals involved in the area of mental health, policy makers, and political decision makers give greater importance to professionally oriented and user-centered facilitating factors. Goal 2 was related to barriers to the usability of emerging technologies for suicide prevention. Both countries and stakeholders attach greater importance to barriers associated with resource constraints than to those centered on personal limitations. There are no differences between countries or between stakeholders. Nevertheless, there is a certain stakeholders-countries interaction that indicates that the opinions on resource constraints expressed by different stakeholders do not follow a uniform pattern in different countries, but they differ depending on the country.

**Conclusions:**

Although all countries and stakeholders agree in identifying resource constraints as the main barrier to the use of emerging technologies, factors facilitating their use in suicide prevention differ among countries and among stakeholders.

## Introduction

The use of emerging technologies involving Internet surfing, virtual social networks, videogames, or mobile phones has led to significant changes in the way people interact, especially the younger population [1]. Applied to health sciences, emerging technologies offer advantages such as easy access to resources and information, personalized medical care, and real-time communication [2,3]. The usefulness of mobile communication technologies in the health area has been known for a number of years [4-7]. Telemedicine has proven effective in various health sectors through the use of apps to treat patients and by facilitating interactions among health professionals [8].

In the area of mental health, the use of emerging technologies has proven effective in the treatment of different mental disorders [9,10], especially anxiety and depression [11,12]. Indeed, interventions in mental health through the Internet have appeared as more cost-effective than traditional interventions [13,14]. However, acceptance by patients and professionals of emerging technologies in the treatment of mental disorders is currently limited, but it could be increased by providing more information about it [15,16]. In any case, it seems clear that these technologies represent an opportunity to supplement many of the treatments carried out in the mental health area by increasing contact and accessibility to therapies, especially for patients living in rural areas and those who usually avoid, for different reasons, mental health facilities [17-19].

Suicide is a serious public health issue, representing one of the main causes of unnatural death worldwide [20]. More than 800,000 people die each year by suicide, the global suicide rate being 11.4 per 100,000 population (15.0 men and 8.0 women) [21]. Although the overall suicide rate in Europe is high, its epidemiology differs considerably among countries [22]. Suicide rates are higher in northern and eastern European countries, the highest being detected in Finland, Hungary, and the Baltic countries, alongside Russia and Belarus, whereas the lowest correspond to southern European countries, such as Italy, Spain, and Greece [23].

It is known that suicidal behaviors are usually preceded by thoughts of death or suicide ideation [24]. Addressing risk factors and an early detection are essential to reduce suicide rates [25]. Currently, there is an awareness of a number of risk factors for suicide, which include neurobiological factors [26], socioeconomic factors [27], and personality traits [28]. Likewise, suffering from or having a history of mental illness and previous suicide attempts are the main risk factors among the general population [29-31]. In this regard, affective disorders, and major depressive disorder in particular, are the mental conditions that involve the highest risk for suicide [32], especially in the elderly [33].

Development of strategies to counteract suicide is one of the priorities of European and worldwide public health systems [34]. In recent years, this has led to the establishment of a growing number of intervention programs in many health care networks [35-37]. However, the stigma associated with mental health care and the difficulties in early detection of the risk for suicide have led to considering the possibility of using emerging technologies to facilitate the access of young people, an at-risk population, to these services while combating the stigma that obstacles help-seeking [38,39].

Suicide prevention programs using emerging technologies [40-42] have also proven effective in the detection of suicide risk [38,43] and in reducing suicide ideation [18,44,45]. Young people look at the Internet also as a useful and accessible tool to express suicidal feelings, seek support, or even try to help other young people having thoughts of suicide [46,47]. This is why technologies such as Twitter, Facebook, forums, text messages, and mobile apps can be of relevance in the area of suicide prevention [48-56]. However, their use is still far from being widespread, and it would be important to be aware of the barriers and limitations that hinder generalized usage.

The purpose of this study was to analyze the potentiality of using emerging technologies in the area of suicide prevention based on the opinion of different professionals involved in addressing this public health issue, as well as possible differences between European countries. The objective was to assess the disposition of professionals to incorporate such resources into the design of a suicide prevention program for the health area of Zamora (Spain). This investigation is encompassed within the European project entitled European Regions Enforcing Actions against Suicide (EUREGENAS), which includes 11 regions with diverse experiences and attempts to advance in the field of suicide prevention in Europe. In particular, we wanted to explore the perception of facilitating factors and barriers related to the use of emerging technologies. So, our goals were as follows:

1. To explore the structure behind the assessment of the facilitating factors for using emerging technologies and compare the relevance attached to them by different countries and stakeholders.
2. To group the barriers to the use of emerging technologies into clusters to determine the importance attached to them and to compare the assessments between countries and between stakeholders.

## Methods

### Participants

Pursuing the aim of efficient intervention in suicide and effective courses of action to be followed for its prevention, a study of the needs at European level was conducted in the context of the Euregenas project. This project brought together 11 European regions with diverse experiences in an attempt to advance the area of suicide prevention in Europe. We wanted to understand the different points of view of those involved in suicide prevention (stakeholders) and the courses of action that could be taken.

First, a consultation with the partners involved in the project and an in-depth review of the literature, and a list of possible stakeholders of interest was proposed. Three main categories of stakeholders were established with different subcategories. The first category corresponded to stakeholders in the political and public management context, designating this category as decision and policy makers (DPM). The second category of stakeholders corresponded to professionals working in the area of mental health, designated as mental health professionals (MHP), and the third one corresponded to professionals related to the social area and nongovernmental organizations (NGOs), designated as NGO/social area (Table 1).

A total of 416 participants were recruited in 11 regions of 8 different European countries according to the following inclusion criteria:

- Workers belonging to the 3 professional groups selected for this study: DPM, MHP, and NGO.
- High professional experience in the field of suicide.
- Age between 18 and 65 years.

### Variables and Instruments

Customized questionnaires including questions on the use of emerging technologies for suicide prevention were prepared for each stakeholder category as tools to gather the necessary information to assess the needs. They included closed questions about the use of emerging technologies for the prevention of suicide. These technologies applied to suicide prevention were defined in the questionnaires as follows: *Technology-based suicide prevention is a form of e-mental health aimed at suicide prevention, making use of information and computer technology*. Some examples of emerging technologies applied to suicide prevention were provided in the questionnaires (Textbox 1).

The sociodemographic data collected in the questionnaires were gender, age, and professional category. The questionnaires were elaborated by some project partners and, subsequently, these questionnaires were revised by all the members of the project. They were drafted in English; each project partner was responsible for translating them into their own language and sending an appropriate number of questionnaires (approximately 60). Questionnaires were mainly administered as face-to-face surveys or via email.

**Table 1:** Categories and subcategories of stakeholders.

Category	Subcategory
Decision and policy makers	European networks focusing on mental health promotion
	Decision and policy makers from local and regional authorities (dealing with mental health, care, welfare, family matters, youth)
	Decision and policy makers in public health institutions (mental health care centers, hospitals)
	Private companies influencing policy (health insurance)
	Media
	Educational setting, policy makers
	Professionals working in financial services and human resources
Mental health professionals (for youth, adult and elderly)	General practitioners
	Psychologists (inpatient, outpatient)
	Psychiatrists (inpatient, outpatient)
	Emergency physicians (on call doctors in Accident and Emergency units)
	Nursing staff who work with suicidal patient (primary health nurse, mental health nurse, emergency room nurse)
	Rescue personnel (paramedic – ambulance crew)
	Work setting (private companies and prevention advisors in occupational medicine)
	Educational setting (schools, school counselors)
Nongovernmental organizations (NGOs)/social area	Professionals in the social area (community social workers, home help workers, youth workers, social welfare services)
	Staff of NGOs and agencies working in the following areas: youth, marital counseling, family and life counseling, welfare
	Educational setting: teachers
	Staff of suicide helplines
	Representatives of religious group
	Support groups with survivors
	Work setting: employers, human resources, union representatives
	Criminal justice stakeholders (police, penitentiary police, coroners)
	Pharmacists

**Examples of emerging technologies applied to the suicide prevention provided in the questionnaires.**

There are many forms of technology-based suicide prevention. Here you can find some examples of what we mean by technology-based suicide prevention:

1. Informative websites (ie, websites that offer information on suicide, including warning signs, risk factors, and what to do when someone is suicidal).
2. Web-based self-help interventions (ie, Web-based interventions that aim at helping (mild to moderate) suicidal people at decreasing their symptoms through self-help).
3. e-therapy interventions (ie, Web-based interventions in which a suicidal person is being guided by a counselor either through a form of self-help in which the counselor is there when needed, or through Web-based and maybe face-to-face therapy).
4. Chat websites (ie, online discussion in a chat room aimed at helping suicidal people through a crisis).
5. Internet forums on suicide and suicide prevention in which suicidal and nonsuicidal people share their thoughts.
6. Social networking websites on suicide prevention (eg, Facebook, Twitter).
7. Apps (ie, apps from the iTunes or Android store on suicide prevention).

In Spain, 154 out of 213 questionnaires facilitated to the stakeholders (72.0 %) were correctly completed. The predominant way of administration was face-to-face (187 questionnaires via face-to-face and 25 questionnaires via email). The most frequent reasons for nonperformance of questionnaires were the incorrect filling of the questionnaire and the absence of a reply by the stakeholder.

Two questions from the survey have been selected for the purpose of this research; both questions aimed at exploring the use of emerging technologies applied to the suicide based on perceived barriers to be removed and facilitating factors to be promoted. These are listed below.

#### Facilitating Factors

What would encourage you to use/recommend suicide prevention based on emerging technologies?

(1=not at all; 5=absolutely):

- Further information through training
- Further information through newsletters
- More automated apps
- Easy access
- Guaranteed anonymity
- Time-saving
- Cost-saving
- Free, with no extra costs

#### Barriers

What prevents you from using/recommending suicide prevention based on emerging technologies?

(1=yes; 0=no):

- Lack of availability
- Too expensive
- Too time-consuming
- Lack of reliable apps
- I do not know their uses
- I am not interested
- I lack the skills
- I lack the knowledge

The questionnaire was administered to a total of 416 participants from 8 European countries. Among this, Spain provided 37.0% of questionnaires; Finland 14.2%; Belgium 11.5%; both Italy and Romania 7.7%; Sweden 7.5%; and lastly, both Germany and Slovenia 7.2% (Table 2).

The gender distribution was 39.7% (165/416) men and 60.3% (251/416) women. According to age, 61.8% (257/416) were aged between 40 to 59 years, 26.9% (112/416) were aged between 20 to 39 years, and 11.3% (47/416) were over 60 years.

### Statistical Analysis

The data analysis of the questionnaires was performed through 2 different statistical methods. First, a multidimensional scaling (MDS) PROXimity SCALing (PROXSCAL) was used to detect the underlying dimensions of the facilitating factors for the use of emerging technologies in suicide prevention. PROXSCAL is a computer program for MDS and individual differences scaling (IDS) of proximities. The program, PROXSCAL, performs MDS of proximity data to ﬁnd the least squares’ representation of the objects in a low-dimensional Euclidean space. The analysis of the structure underlying the usability barriers of emerging technologies for suicide prevention was performed through hierarchical cluster analysis. Finally, multivariate analysis of variance (MANOVA) was used to estimate the differences between countries and stakeholders. The data analysis was performed using the statistical software IBM SPSS version 19.

**Table 2:** Questionnaires administered by country.

Country	DPM^a^	MHP^b^	NGO^c^	Percentage (%)
Belgium	14	19	15	11.5
Finland	7	21	31	14.2
Germany	9	9	12	7.2
Italy	10	13	9	7.7
Romania	10	19	3	7.7
Slovenia	10	11	9	7.2
Spain	17	92	45	37.0
Sweden	10	13	8	7.5
Total	87	197	132	100

^a^DMP: decision and policy makers.

^b^MHP: mental health professionals.

^c^NGO: nongovernmental organizations.

## Results

### Goal 1: Factors Facilitating the Use of Emerging Technologies

MDS PROXSCAL was applied to identify the underlying dimensions of facilitating factors for using emerging technologies in suicide prevention. This analysis provides a display of how the facilitating factors are structured and their weight in the different target populations.

The starting point is the exploration of a common space that reveals a structure in the assessments of the interviewees. If a common structure is found, the underlying criteria used for the assessments can be identified, which are not explicit, but deducible from the way in which the assessments accorded to the facilitating factors relate to each other. This technique is useful to identify the criteria used when assessing the aspects related to the use of emerging technologies. In this case, beyond specific assessments for each aspect, the interest lies in exploring the underlying criteria so that the actions to stimulate the use of emerging technologies can be linked to the aspects that are most relevant to each group of the target population.

**Figure 1:**
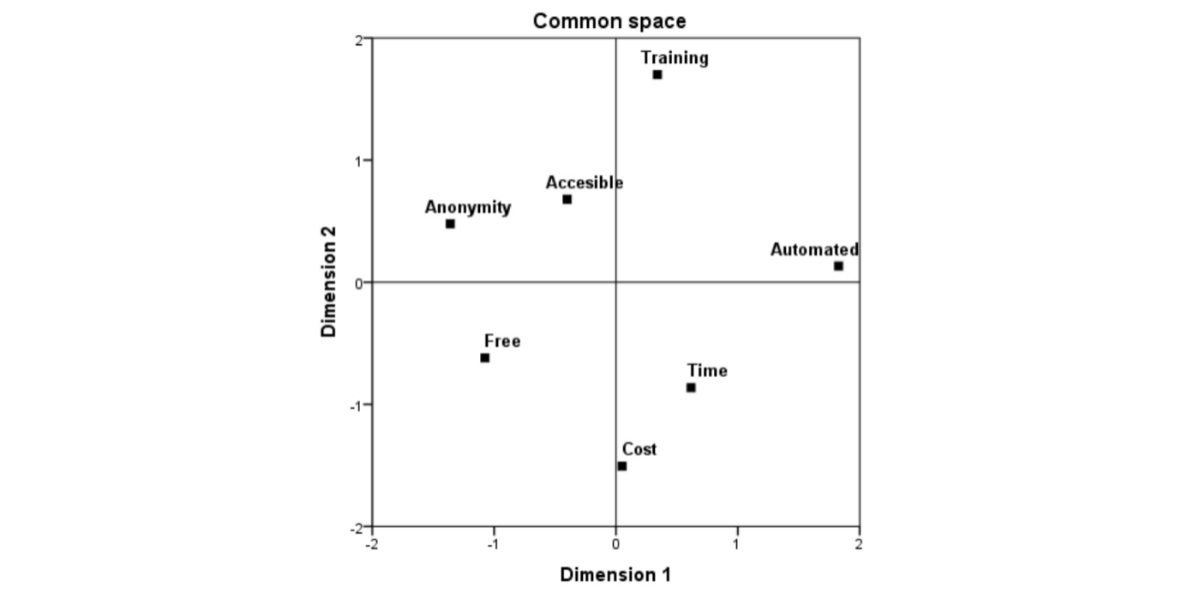
Common space of the facilitating factors in the multicountry sample.

The perceptual map resulting from this analysis reveals that there is indeed an identifiable structure the indices of which indicate a very good fit to the empirical data. A scatterplot matrix of coordinates of the common space is displayed in Figure 1. The structure presents a good fit to the obtained dimensions (Stress=.05; Tucker's Coefficient of Congruence=.973>.90).

As shown in Figure 1, Dimension 1 on the right-hand area of the chart brings together aspects related to cost for professionals: training costs (*Training*), cost in time (*Time and Automated*), and economic cost (*Cost*). At the opposite end are the facilitating factors related to aspects that are convenient for the user, which are as follows: *Anonymity*, *Accessible*, and *Free*. Therefore, Dimension 1 opposes professionally oriented facilitating factors and user-centered facilitating factors.

On the right-hand upper area of the graph, Dimension 2 brings together the aspects related to the implementation of emerging technologies, which are as follows: *Training*, *Anonymity*, *Accessible*, and *Automated*. The lower area includes facilitating factors related to the benefits from their use, which are as follows: *Cost*, *Time*, and *Free*. Therefore, Dimension 2 opposes facilitating factors that optimize implementation and facilitating factors that optimize the benefits.

Thus, the following 2 dimensions can be identified in the common space:

- Dimension 1: professionally oriented facilitating factors and user-centered facilitating factors.
- Dimension 2: facilitating factors that optimize implementation and facilitating factors that optimize the benefits.

**Figure 2:**
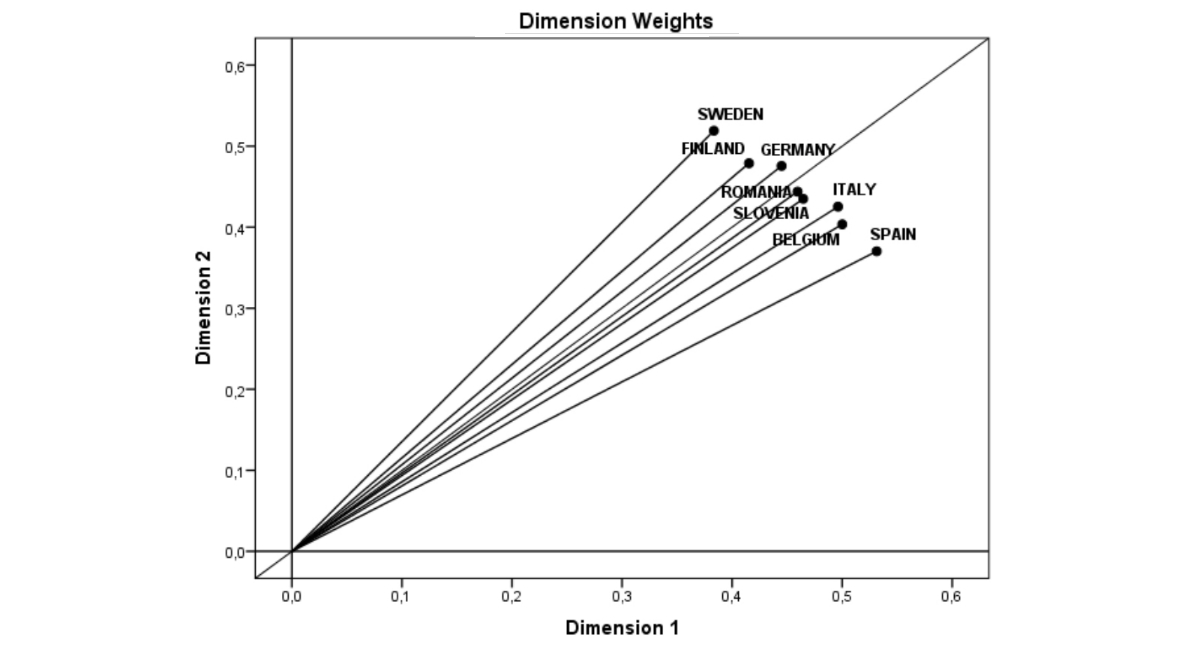
Dimension Weights of the facilitating factors for suicide prevention in the different countries.

Once the structure or common space has been defined, the weight of each dimension in the different target populations is obtained. In this case, the weight or relevance of each dimension was obtained for different countries and different stakeholders. Figure 2 shows the different weights for the different countries studied.

As shown in the chart, Dimension 2 (implementation-benefits) has greater weight than Dimension 1 (professional-user) in Sweden, Finland, and Germany, in that order. Thus, in these countries, emphasis should be placed on the adaptation of the characteristics involved in the implementation of emerging technologies (*Training*, *Accessible*, *Anonymity*, and *Automated*) and on the variety of benefits they provide (*Cost*, *Time*, and *Free*) because they attach higher relevance to these criteria in their assessments. On the other hand, in Spain, Belgium, Italy, Slovenia, and Romania, in that order, Dimension 1 (professional–user) has higher weight than Dimension 2 (implementation–benefits). Thus, it is appropriate for these countries to emphasize the benefits to be obtained by the use of emerging technologies, both for professionals and for users.

**Figure 3:**
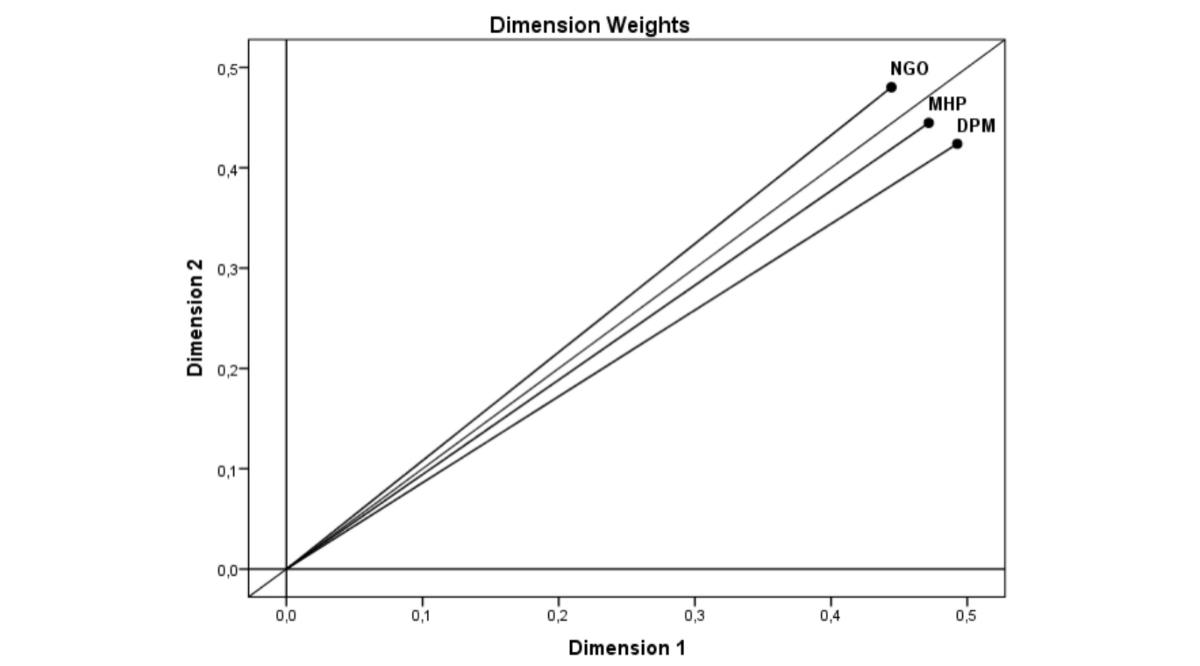
Dimension weights of the facilitating factors in the different stakeholders.

The comparison of weights by stakeholders also reveals differences in the relevance attached to these 2 dimensions, as shown in Figure 3.

NGOs attach greater weight to Dimension 2 (implementation–benefits) than to Dimension 1 (professional–user). To promote the use of emerging technologies by NGOs and achieve greater usability, emphasis should be placed on the conditions for the implementation of such resources and the benefits they bring. On the other hand, DPMs and MHPs, in that order, attach greater relevance to Dimension 1 (professional–user) than to Dimension 2 (implementation–benefits). To promote the use of emerging technologies by these stakeholders and achieve greater usability, emphasis should be placed on the aspects related to the benefits they bring to both professionals and users.

### Goal 2: Barriers to the Use of Emerging Technologies

**Figure 4:**
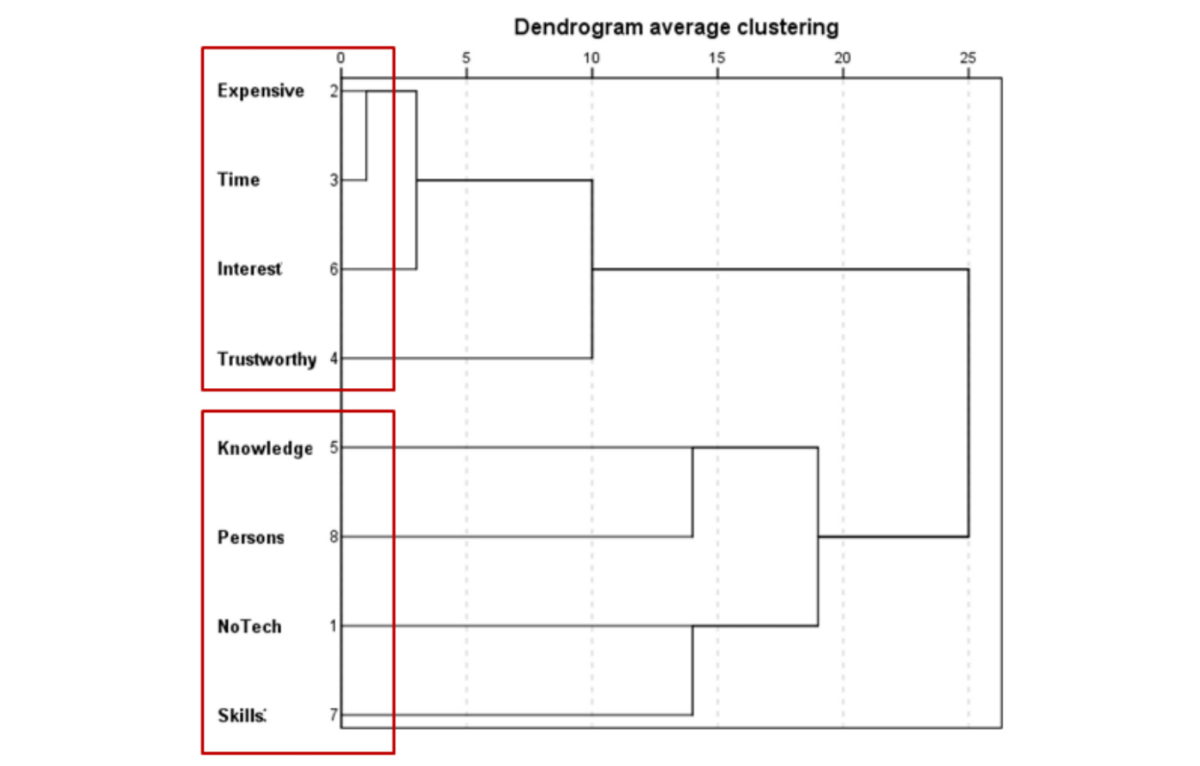
Clusters of barriers to the use of emerging technologies.

The analysis of the structure underlying the barriers hindering the use of emerging technologies for suicide prevention was carried out through hierarchical cluster analysis (Figure 4). As shown in the chart, the barriers were gathered into the following 2 large groups or differentiated clusters:

- Barriers focused on resource constraints: expensive, time-consuming, not interesting, and apps are not considered trustworthy.
- Barriers focused on personal limitations: lack of knowledge related to the programs and their use and lack of skills to use them.

The average ratings for each cluster were calculated to compare the ratings obtained and the differences among countries and stakeholders. A repeated-measure analysis of variance (ANOVA) was carried out. The results show significant differences among clusters (*P<*.001) with large effect size: 44%. Cluster 1 (barriers focused on resource constraints) scored significantly higher than cluster 2 (barriers focused on user limitations).

Therefore, to promote the use of emerging technologies for suicide prevention, emphasis should be placed on the shortcomings of the resource itself, meaning that difficulties are attached to resource constraints rather than to users experiencing difficulties.

**Figure 5:**
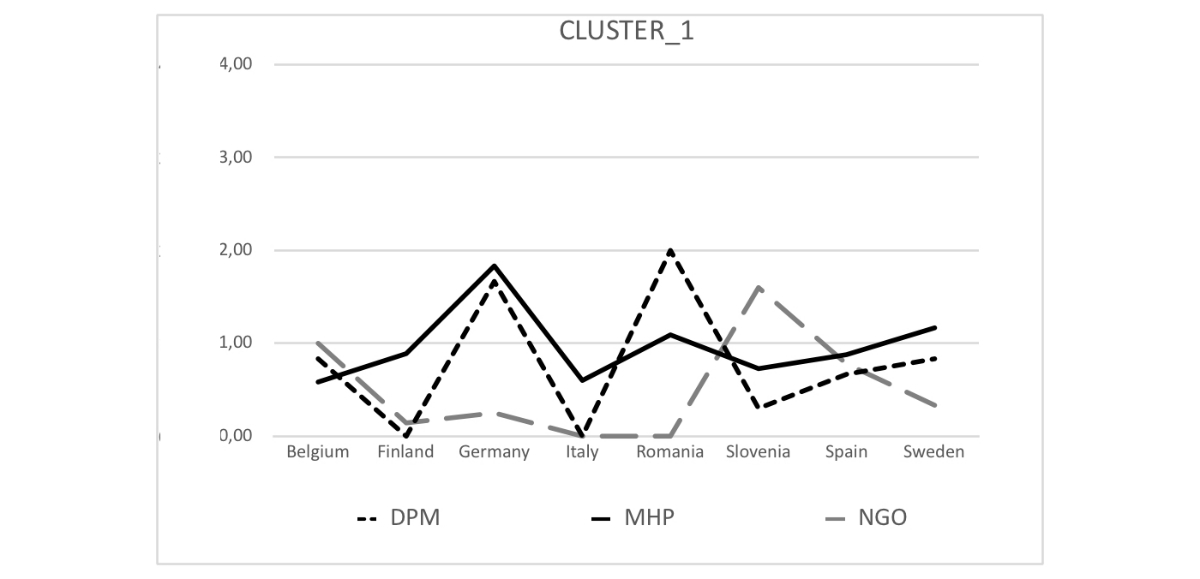
Cluster 1: barriers focused on resource constraints.

Differences among countries and stakeholders were calculated using MANOVA. No differences among countries were observed (Pillai Trace Test: *P=*.86); barriers focused on resources were those that most hindered implementation in all of them. Neither were there differences among stakeholders (Pillai Trace Test: *P=*.08); all of them also attached the most relevance to barriers related to resources. Nevertheless, stakeholders-countries interaction effect (Pillai Trace Test: *P=*.01) was observed in cluster 1; barriers focused on resource constraints (*P*=.02) with an effect size of 13%. This interaction effect indicates that the valuations of resource constraints made by the different stakeholders do not follow a standard pattern in the different countries, but they are different depending on the country, as shown in Figure 5.

As shown in the graph, in Germany, for example, MHPs are the stakeholders that attach the greatest importance to resource constraints, whereas in Romania, it is DPMs, and in Slovenia, it is NGOs. In Spain, for example, the valuations of the different stakeholders are very similar. Each country has different profiles with regard to resource constraints, which should be taken into account according to the target stakeholders at whom promotion of the use of emerging technology for suicide prevention is aimed.

## Discussion

### Principal Findings

The use of emerging technologies for suicide prevention could prove an opportunity to ameliorate its results and the accessibility to intervention programs, especially by young people. Emerging technologies can be a means to address situations involving people at suicide risk who are otherwise difficult to engage in traditional intervention models. A recent study by Sueki supports the efficacy of the use of Internet for early detection of suicide risk. The study involved posting offers of psychological treatment on webpages that people at risk for suicide might visit. The psychological treatment was provided via email, and the presence of suicide ideation was detected in 74% of the consultations held [43]. Therefore, it improves accessibility to a population that does not usually turn to health services, and when they do so, they have already made a suicide attempt [57].

If we consider the factors facilitating the usability of emerging technologies according to the different countries, it is interesting to observe how northern European countries, except for Belgium, attach greater importance to the facilitating factors that optimize implementation and to those than maximize the benefits (Dimension 2) than to those focused on professionals and users (Dimension 1). To some extent, this result could be explained by the differences between northern and southern European countries in the use of emerging technologies, considering that the implementation of emerging technologies in northern Europe is more widespread than in the south. On this subject, when defining the type of intervention, northern European countries seem to attach more importance to ensuring efficacy than to it being different or more or less innovative. On the other hand, southern European countries seem more concerned with the development of the intervention than with its degree of efficiency. In this regard, the importance attached in Spain to barriers and difficulties hindering professionals’ use of this type of technologies can be observed. This basically expresses that the use of emerging technologies involves changes in traditional welfare processes based mainly on routine clinical visits and scheduled appointments. It is also significant that in countries where psychiatric care is essentially community-based and health care has a solid social component, as is the case in Sweden, emphasis on barriers and facilitating factors centered on users and professionals is not as strong as that placed on the implementation and efficacy of the tool. This leads to the hypothesis that the use of emerging technologies to contact users or potential patients requires a more community-based health system, which is less constrained by clinical visits, and where the measuring of health care activities goes beyond a mere count of the number of medical consultations performed following conventional methods. How could the activity conducted by MHPs communicating with patients via social networks be calculated and measured? Or, could the Spanish system for the provision of posts envisage establishing a profile for MHPs with expertise in the use of emerging technologies to allow potential patients to access their services? Hence, the answers to the questionnaire provided by the different countries also reveal their care patterns and their capacity to integrate the use of new models, such as the use of emerging technologies, into the dynamics of their activity.

Likewise, although the stakeholders participating in the study are aware of the advantageous uses of emerging technologies for users, they stress the difficulties associated with their use by professionals and the costs in time, training, and activity involved in their implementation. This is the main barrier to their use, as there is an agreement (Dimension 2) on valuing the positive aspects related to accessibility and anonymity so that the user does not feel exposed to stigmatization.

On the other hand, the distribution of the 3 different stakeholder groups across the two dimensions draws attention to the fact that professionals in the social area (NGO) stand apart from the other 2 groups (DMP and MHP). The former (NGO) attach greater relevance to the facilitating factors that optimize implementation and to those that maximize the benefits (Dimension 2), whereas the other groups (DPM and MHP) believe that the most relevant facilitating factors are the professionally oriented and user-centered ones (Dimension 1). These results could answer to the usual need for professionals in the social area to obtain positive outcomes in their health interventions, because negative results or absence of results have a very strong impact on this group. It should be noted that when the health system fails in its interventions, this has a strong impact on the subject’s social environment, which is often poor or lacking adequate supporting structures, thus requiring the intervention of social services. MHPs believe that the most important facilitating factors are those that are professionally oriented and user-centered (Dimension 1), although to a lesser degree than DMPs. This confirms what is stated above, that is, public-sector providers whose health care processes are more constrained and move away from the community-based ones are those who find the greatest difficulties in using emerging technologies. So, they do not take enough account to use the technologies as a tool for improving access to better care. It is significant that the further stakeholders from the community (political decision makers and policy makers) give a higher value to the barriers to their work, while the closer to the community underlie the greatest flexibility for organizing the delivery of care (NGOs), and increasing the efficacy that technology could make, while the difficulties encountered by professionals are given less relevance.

In general, although emerging technologies are a tool that can be used to address problems related to suicidal behavior, the knowledge required to design their use and obtain satisfactory results belongs to the MHPs. This explains why MHPs are more concerned with the elements that may facilitate implementation of the intervention than with its costs, even though they are aware of the limitations of the system of which they are part. Finally, the fact that political decision makers and policy makers (DPM) attach greater relevance to facilitating factors targeted at professionals and users (Dimension 1), leaving aside the economic aspect, is highly significant, because it illustrates the extent of the problem of suicide in the Western society and the increasing involvement of this group of stakeholders in addressing suicidal behavior. Nevertheless, they should be aware that, beyond the application of such technologies, it is essential to foster working on the community and directing (mental) health services toward (mental) health outcomes in the population of the area where they are located, rather than just measuring health care–oriented activity in a rigid fashion where there is no room for these new tools.

The use of technology applied to the health care area does not always yield expected or desirable results, but limitations or barriers may appear. However, in the area of suicide prevention, this type of communication technologies could provide major advantages, because limitations to intervention in these cases are frequently linked to the stigma attached to the issue of suicide in our society. Good examples of clinical uses of technology for the suicidal behavior are as follows: Mewton et al’s work, which implemented a Web-based program of cognitive behavioral therapy to reduce suicidal ideation in people suffering from depression, obtaining statistically significant results in the reduction of self-harming ideation and symptoms of depression in general terms [18]; and Guille et al’s work, which refers to an ease of implementation of Web-based cognitive behavioral therapy, emphasizing that it is cost free and user-friendly and very useful for suicide prevention [41].

Consequently, the growing deployment of Internet in our environment and its development as a way of communication offers an excellent chance to use it as a means for the detection and treatment of suicidal behavior and ideation. The possibility of distance intervention that does not require contact in a physical space is an advantage for the high number of individuals who perceive the issue of suicide as a taboo. In a recent study, Biddle analyzed the changes in accessibility to suicide-related information on the Internet between 2007 and 2014, obtaining as a result that the number of blogs and Internet forums on suicide grew considerably over this 7-year period [46]. This is why it is important to assess the results obtained and take them into account to be able to overcome the barriers to the implementation of emerging technologies in the health system.

A key aspect to be considered in the application of the emerging technologies to health care is the anonymity. This anonymity can be appreciated clearly in the Internet use by persons who look for information about issues relating to health. It should be pointed out that the use of Internet browsers such as Google is currently widespread in our environment, and the trend analysis of search for research purposes have grown in recent years. For its capacity to ensure anonymity, these Internet browsers can be used by persons with thoughts or ideas of suicide to search for help online. On the other hand, Internet browsers might also constitute a useful tool for the study and detection of behaviors related to suicide [58,59]. There are several studies that have used the potential of the Google Trends tool in the field of suicide and self-harm behaviors. One study by Bragazzi shows the usefulness of Google Trends to detect nonsuicidal self-injuries [60]. Another recent study by Parker could verify that the use of Google Trends can predict the suicide rate associated with the consumption of alcohol and drugs better than the conventional methods associated with the level of unemployment and economic incomes [61]. Solano detected that the search volume of the term “suicide” is significantly related with the suicide valuations in Italy [62], and Arora observed a cyclical tendency in the search activity of suicide and in the searches related to the depression, with peaks in autumn and winter months and a decrease in summer months [63].

Equally, attention should be drawn to the fact that the analysis of the results of this study on the barriers to the use of emerging technologies shows that barriers focused on resource constraints (cluster 1) are more relevant, both by countries and stakeholders, than those focused on personal limitations (cluster 2). Once again, this points out the difficulties in organization and in obtaining resources for the implementation of these new tools. The use of new technologies in the health sector requires appropriate organization and a care delivery model to facilitate their implementation, economic resources to acquire the material needed to build the tool’s structure (hardware), and the availability of skilled technicians to update and develop the functioning of the structure (software). For this reason, it should be noted that, despite the fact that, in the mid and long term, intervention based on emerging technologies could prove more efficient than traditional methods, these resources are not always available, sometimes not at all. This would also justify the limitations observed for emerging technologies to become integrated into the health system, an example of this being telemedicine, the implementation of which is taking place at a much slower pace than expected [64].

In this regard, Donker’s recent systematic review to assess the cost of Internet-based mental health interventions proved their greater cost-effectiveness [13]. Van Spijker conducted a randomized controlled clinical trial to analyze the cost-effectiveness of Web-based interventions for the reduction of suicidal ideation, and it was found that they were indeed more cost-effective than traditional interventions [14]. This means that there are advantages in terms of effectiveness and costs and that it is a relevant public health issue, which leads to the question of why their use is not more widespread.

Finally, solely considering the barriers focused on resource constraints (cluster 1), it can be observed that there are differences among countries and stakeholders. The most remarkable cases are Germany and Romania, where the differences among the different stakeholder groups are stronger, as opposed to the rest of countries, where there is greater consistency among the 3 stakeholder groups. Nevertheless, these differences are mainly associated with barriers observed by health professionals rather than managers, and it is necessary to study such differences in depth to assess the reason for their existence, which could perhaps be attributed to shortcomings related to the sample and should be confirmed in future studies.

Attention should also be drawn to limitations in our sample, which, though randomly selected from each country based on the subjects’ experience in the area of suicide, is not representative of the entire group it is part of. Nevertheless, it is adequate for a first approach to the study of limitations to the use of technologies in the health care area, assessing contrasts among countries and differences in health care models. On the other hand, the heterogeneous distribution of the different professional categories taking the survey makes it difficult to ensure a representative sample for each of them. Still, the data gathered contribute an interesting approximation to the potential and facilitating factors and barriers involved in the use of emerging technologies for the prevention and treatment of suicidal behavior.

### Limitations and Strengths

The stakeholders involved in the study were not selected in a randomized way; therefore, they are not representatives of the stakeholders as a whole. The number of stakeholders involved in the study is different in every country, and probably, the motivation for answering is different too. Besides, the sociodemographic data collected in the questionnaire (gender, age, and professional category) could have an impact on the findings, but it has not been possible to control these variables because of the small size of this study sample. It should be considered that the principal objective of the project was to analyze the knowledge of relevant professionals in suicide field to improve and create prevention programs of suicide in different regions of Europe. The questionnaire used to collect the data was elaborated internally by the members of the project, and this questionnaire was not validated regarding the psychometric criteria. It should be highlighted that the questionnaire was not designed like a useful tool in the prevention of the suicide and was elaborated just for the compilation of the data. The different translations of the questionnaire into each language of the country were not made in a homogenous way; every project partner made the translation from English using different translation resources.

Taking into account these limitations, the differences between countries can be associated to different perspective of the specific stakeholders selected instead of proper general differences between countries. However, the data are interesting for knowing the possibilities and potential benefits of technologies for being used in suicide prevention. In this sense, it is the first study in Europe comparing different countries (south-north/east-west) regarding this topic.

### Conclusion and Clinical Implications

There is evidence that new communication technologies may help toward improving suicide prevention although their implementation and use in the health system is still quite limited. Barriers to their use are different from one country to another and also depend on the organizational models. Equally, assessments vary depending on professionals consulted. Southern European countries, such as Spain, where health care models are more traditional and not community-based, instead of focusing on the effectiveness and advantages that this new type of health care model could contribute, believe the main barriers are related to the organizational system, the characteristics of the health professionals, and the difficulties they experience when using emerging technologies for this purpose. In contrast, countries such as Sweden, with community-based health care models and, therefore, a more flexible organization that facilitates the implementation and use of these technologies, consider that the main difficulties lie in proving their effectiveness in the delivery of services and ensuring that they actually facilitate accessibility.

On the basis of the results of this study, we consider that a broader use of communication technologies in suicide prevention would facilitate accessibility and care of people at risk for suicide. However, to apply these tools it is necessary to change organizational models, taking into account both the investment required and the changes in health care provision, which should be more flexible and targeted at results rather than at specific activities (eg, medical consultations). This is probably one of the main obstacles that has so far limited the implementation of emerging technologies.

## Abbreviations

ANOVA: analysis of variance
DPM: decision and policy makers
EUREGENAS: European Regions Enforcing Actions against Suicide
IDS: individual differences scaling
MANOVA: multivariate analysis of variance
MDS: multidimensional scaling
MHP: mental health professionals
NGOs: nongovernmental organizations
PROXSCAL: PROXimity SCALing
